# Evaluating the diet in Germany with two indices focusing on healthy eating and planetary healthy eating using nationwide cross-sectional food intake data from DEGS1 (2008–2011)

**DOI:** 10.1007/s00394-024-03476-x

**Published:** 2024-08-15

**Authors:** Almut Richter, Julika Loss, Daria-Alina Kuhn, Ramona Moosburger, Gert B. M. Mensink

**Affiliations:** Department of Epidemiology and Health Monitoring, Robert Koch Institute, Berlin, Germany

**Keywords:** Healthy eating index, Planetary healthy eating, EAT-Lancet, Nutrition, Climate, Representative sample

## Abstract

**Purpose:**

To improve sustainability, adjustments to current diets are necessary. Therefore, limited planetary resources are considered within the healthy reference diet proposed by the EAT-Lancet Commission. The agreement with nationwide food intake was evaluated with two indices which reflect this reference and German food intake recommendations.

**Methods:**

A healthy eating index (HEI-MON) reflecting the dietary guidelines of the German nutrition society and a planetary healthy eating index (PHEI-MON) reflecting the healthy reference diet were developed, with scores from 0 to 100. Both indices were applied to data from a nationally representative sample of the German population aged 18–79 years for which data from a 53-item food frequency questionnaire are available.

**Results:**

Mean scores for the indices were 53 for HEI-MON and 39 for PHEI-MON. A better adherence to either guideline could be found among women, persons of older age as well as persons with higher education level. The sub-scores for HEI-MON showed high agreement with the recommendations for side dishes, fruit/nuts, (processed) meat and cereals, but low agreement with the recommendations for free sugar and vegetables/legumes. PHEI-MON sub-scores were highest for poultry, fruits and potatoes, and lowest for nuts, red meat and legumes. High scores in one index do not necessarily correspond to high scores in the other index. Individuals with more plantbased diets had higher scores in both indices, while high sugar and meat consumption led to lower scores.

**Conclusions:**

More plant-based diets are crucial for individual and planetary health. Both indices reflect such diets which consider already health and sustainability aspects. At an individual level, the scores for both indices may differ considerably, but overall there is a huge potential in the population to adapt to a diet more in line with both guidelines.

**Supplementary Information:**

The online version contains supplementary material available at 10.1007/s00394-024-03476-x.

## Introduction

Food based dietary guidelines (FBDG) outline a diet which provides adequate energy and nutrients and has a positive impact on health and life expectancy. To facilitate adherence to these guidelines, they should account for prevailing dietary habits in the target population. Considering the climate crisis, limited resources and planetary boundaries [1], the EAT-Lancet Commission published a reference diet which covers both health and sustainability aspects [2]. FBDG in Germany currently concentrate on an adequate and health promoting diet [3], although sustainability aspects of diet will be given more priority in the near future.

Currently, food systems are worldwide responsible for about 19–29% of climate-relevant greenhouse gas emissions [4]. The common dietary habits and food systems in Germany, like in many high-income countries, may have negative impacts on long-term food supply for a growing world population, and may also contribute to global warming and affect planetary as well as individual health [2]. This development may be counteracted if many people follow dietary guidelines which foster both health and sustainability.

FBDG usually consist of several recommendations on different food components. Tools like indices, which summarize the alignment with these recommendations, may be useful for monitoring purposes. The development and application of a healthy eating index can help to evaluate the extent to which a person’s diet meets the FBDG [5]. Such an index can also be based on guidelines which include sustainability aspects, like the EAT Healthy Reference Diet (EAT-HRD) [2]. Although differences between national FBDG and the EAT-HRD have been shown in several countries [6–8], the EAT-HRD has also substantial overlaps with common FBDG, like the Dietary Guidelines for Americans and the FBDG of the German Nutrition Society [6, 9], but differs, among others, in preferred protein sources. In comparison to the German FBDG it recommends lower intakes of dairy products and gives separate recommendations for red meat, nuts and legumes. To date, several studies have already constructed indices reflecting the EAT-HRD [10–20] but only a few have applied them to representative population samples [10–12, 18]. Dietary data for these analyses were obtained using different assessment methods, such as 24-h recalls [8, 11–13, 18, 20] or a food frequency questionnaire (FFQ) [14, 15, 17, 19, 21–23]. Partly because of these differences in data, different scoring systems have been used. While a few indices used a gradual scoring [13, 18, 19, 23, 24] or scores from 0 to 3 points [22, 25, 26], most indices are based on dichotomous ratings (0 or 1 point) [11, 12, 14–16, 21, 27].

Common dietary habits may vary considerably between countries. The development and use of these country specific indices allow a holistic view to evaluate dietary intake in the specific population and to identify differences in certain subgroups. The indices can also be used to analyse associations between usual diet and health outcomes. To evaluate dietary habits in Germany, we used FFQ data from a German national health survey (German Health Interview and Examination Survey for Adults, DEGS1) to construct two indices: a healthy eating index—for monitoring (HEI-MON) reflecting the alignment with the FBDG of the German Nutrition Society (GNS) [9] and a planetary healthy eating index—for monitoring (PHEI-MON) reflecting the EAT-HRD, partially customized to German diets. For both indices we adapted a previously developed scoring system [28], with a very differentiated, gradual scoring in which 0–100 points are awarded. The concept of the PHEI-MON is partly similar to a previously published Planetary Health Diet Index (PHDI) [19], which also uses a gradual scoring system, however with 0–10 points. Since our indices are based on a fairly short FFQ they may be conveniently applied within a health monitoring system.

We applied both indices to evaluate the diet of people living in Germany based on DEGS1 data. By comparing the results for both indices, we addressed the following questions:

- To what extent do German dietary habits align with both recommendation sets?
- Are there differences between population subgroups, e.g. in terms of gender, age or education level?
- Which components of the indices (or food groups) have especially high or low scores?
- What proportion of people with a high score on one index also have a high score on the other index?
- Which differences in food intake can be seen for those who score high on only one index and not on the other, or those who score high on both indexes?

## Materials and methods

### Study design and sample

DEGS1 is a nationwide cross-sectional representative health survey among 7987 adults aged 18–79 years, conducted between 2008 and 2011. The design and methods of DEGS1 have been described previously [29]. In short, participants of the previous German National Health Interview and Examination Survey 1998 (GNHIES98) [30] were re-invited. In addition, persons were recruited for DEGS1 in 2008–2011 based on two-stage stratified random sampling from local population registries [29]. The net sample included 3959 re-invited and 4193 new participants, aged 18–91 years, of which 7987 aged 18–79 years. Among those aged 18–79 years, 7115 participants attended a one-time physical examination and received a single semi-quantitative food frequency questionnaire (FFQ; paper/pencil) several days prior to their visit to the study centre and were asked to bring the completed questionnaire to the appointment. This FFQ was validated among 161 adults who completed two independent standardised 24-h dietary recalls in addition to the FFQ. The ranking of intake quantities per food item in the same or adjacent quartile for both methods ranged between 68% for cooked vegetables and 94% for coffee, which implies a reasonable to good validity [31].

In the FFQ, the question “How often did you eat (or drink)…?” was asked for 53 food items (referring to the last four weeks). The intake frequencies could be answered with the categories “never”, “once a month”, “2–3 times a month”, “1–2 times a week”, “3–4 times a week”, “5–6 times a week”, “once a day”, “2 times a day”, “3 times a day”, “4–5 times a day” or “more often than five times a day”. The portion amounts were obtained with food-specific answer categories, reflecting generally “½ portion (or less)”, “1 portion”, “2 portions”, “3 portions” or “4 portions (or more)” as well as—depending on the food—“¼ portion”. Standard portion units were given, depending on the food item, for example: glass, cup, mug, bowl, plate, slice or piece. In addition, photos were included in many questions to illustrate the standard portion sizes. To calculate the average daily intake of a food (group) in grams per day, the intake frequency was multiplied by the corresponding portion amount and divided by 28 days (intake frequency * portion amount (g)/28 days).

A total of 7079 FFQs were completed. After plausibility checks, 70 participants were excluded from the analysis because their questionnaires were incomplete (more than 20 missing frequency values; n = 8) or had implausibly high or low intakes (n = 62). Very high consumption quantities (mean per day) were considered implausible in nine participants (more than 15 L of beverages or more than 10 kg of solid food or more than 4 L of beverages and at the same time more than 6 kg of solid food) as well as very low consumption quantities in 53 participants (less than 200 ml of drinks; less than 200 g of solid foods; solid foods and drinks together under 1 kg and simultaneously more than 20 missing portion sizes; solid foods and drinks together under 500 g).

Accordingly, 7009 participants were included in the analysis. If both, the intake frequency and the corresponding portion information were missing, the specific food was considered “not consumed”. If the intake frequency answer was available but the portion amount was missing, it was replaced by the median portion category of the respective food item as determined for all participants.

### Healthy eating index—for monitoring (HEI-MON)

The HEI-MON developed for monitoring dietary patterns within DEGS1 uses a similar scoring system as the healthy nutrition score for kids and youth (HuSKY) developed for the KiGGS study FFQ [28]. To construct the HEI-MON, food items of the FFQ (SI 1) were grouped into ten food groups (Table 1). Individual food intake was rated based on the FBDG of the GNS [3, 9]. For each food group a score for adherence to the specific recommendation was calculated using the ratio of the obtained to the recommended intake amount. Based on this ratio, the degree of adherence to the respective recommendation was awarded with a maximum of 100 points for each food group. For the allocation of points, different rules were applied, depending on the recommendation to eat plenty, optimal or sparingly amounts of the specific food group. For foods to be consumed in plenty amounts, the score increases with a higher intake because a higher intake is assumed to have beneficial health effects. For foods to be consumed in optimal amounts, less points are assigned with intake quantities above the recommended amounts, since a high intake of the corresponding foods is associated with adverse effects on health. Sugar should be consumed sparingly, therefore 100 points are given for an estimated zero intake (Table 1). In addition, for all food groups except meat/processed meat and free sugars points are deducted if the quantity consumed falls below a certain amount. For example, an intake equal to 50% of the recommended quantity received 50 points.

**Table 1:** Construction of the healthy eating index—for monitoring (HEI-MON) and the planetary healthy eating index—for monitoring (PHEI-MON): food groups, FFQ-items, intake recommendations, evaluation principles and scoring

		FFQ-items^a^	Recommendation^b^	Rule for point allocation	Scoring 0–100 points, depending on the amount consumed			
					100 points	Points are reduced proportionately	Points are reduced proportionately	0 points
Healthy eating index—for monitoring (HEI-MON)	Cereals/bread	8, 9, 10, 11, 12	200–310 g/day	Optimal	200–620 g	< 200 g	> 620 g	> 930 g
	Side dishes (potatoes, pasta, rice)	32, 33, 34, 35, 36	150–250 g/day	Optimal	150–500 g	< 150 g	> 500 g	> 750 g
	Vegetables/legumes	4, 29, 30, 31	3 portions/day^c^	Plenty	≥ 3 portions	< 3 portions		0 portions
	Fruits/nuts	3, 27, 28, 43	2 portions/day^d^	Plenty	≥ 2 portions	< 2 portions		0 portions
	Milk/dairy products	1, 13, 15	200–250 g/day	Optimal	200–250 g	< 200 g	> 250 g	> 500 g
	Cheese	14	50–60 g/day	Optimal	50–60 g	< 50 g	> 60 g	> 120 g
	Eggs	18	26 g/day	Optimal	26 g	< 26 g	> 26 g	> 52 g
	Fish	25, 26	21–31 g/day	Optimal	21–31 g	< 21 g	> 31 g	> 62 g
	Meat/processed meat	19, 20, 21, 22, 23, 24	If any 43–86 g/day	Optimal	0–86 g		> 86 g	> 172 g
	Free Sugar	2, 3, 4, 5, 6, 7, 16, 17, 37, 38, 39, 40, 41	0–60 g/day^e^	Sparingly	0 g		> 0 g	> 60 g
Planetary healthy eating index—for monitoring (PHEI-MON)	Whole grains	9, 10	0–60% of energy intake	Optimal	≥ 15–60%	< 15%	> 60%	100%
	Potatoes	34, 35, 36, 42	50 (0–100) g/day	Optimal	50–100 g	< 50 g	> 100 g	> 200
	Vegetables	4, 29, 31	300 (200–600) g/day^f^	Plenty	≥ 300 g	< 300 g		0 g
	Fruits	3, 27, 28	200 (100–300) g/day^f^	Plenty	≥ 200 g	< 200 g		0 g
	Nuts^g^	43	50 (25–100) g/day	Plenty	≥ 50 g	< 50 g		0 g
	Legumes^h^	30	143 (0–268) g/day	Plenty	≥ 143 g	< 143 g		0 g
	Milk equivalents^i^	1, 13, 14, 15	250 (0–500) g/day	Optimal	250 g	< 250 g	> 250 g	> 500 g
	Red meat	20, 21, 22, 23, 24	14 (0–28) g/day	Sparingly	0 g		> 0 g	> 28 g
	Poultry^j^	19	29 (0–58) g/day	Optimal	0–29 g		> 29 g	> 58 g
	Eggs	18	13 (0–25) g/day	Optimal	13 g	< 13 g	> 13 g	> 25 g
	Fish	25, 26	28 (0–100) g/day	Optimal	28 g	< 28 g	> 28 g	> 100 g
	Added sugars	2, 5, 6, 7, 16, 17, 37, 38, 39, 40, 41	31 (0–31) g/day	Sparingly	0 g		> 0 g	> 31 g

^a^For food frequency questionnaire items see SI 1

^b^HEI-MON based on the German Food Based Dietary Guidelines of the German Nutrition Society [3, 9], PHEI-MON based on the healthy reference diet of the EAT-Lancet Commission[2]

^c^One portion of legumes is 125 g, one portion of vegetables is 133 g, up to one glass of vegetables juice was included in the amount vegetables consumed

^d^One portion of nuts is 25 g, one Portion of fruits is 125 g, up to one glass of fruit juice was included in the amount fruits consumed

^e^In the German food based dietary guidelines (FBDG) there is no specific recommendation for sugar intake, therefore the tolerated amount was derived from another source [53] and corresponds to a maximum of 10% of the total energy. For a total energy intake of 2400 kcal this is 60 g free sugars [9]

^f^Up to one glass of juice was included in the amount consumed

^g^This food group includes tree nuts and peanuts

^h^Dry weights of these food group were converted to consumed amounts with a factor of 2.86 according to [6]

^i^Dairy products were converted to milk using the following factors: item 15: factor 3; item 16: factor 7; item 17: factor 2

^j^Poultry can be exchanged by fish and/or eggs

### Planetary healthy eating index—for monitoring (PHEI-MON)

To calculate the PHEI-MON, FFQ items (Table 1) were grouped according to food groups addressed in the EAT-Lancet publication [2]. This includes a group for whole grains, to which we assigned whole grain versions of cereals and bread. Information on whole grain versions of rice or pasta was not assessed and could therefore not be included. The EAT-HRD includes potatoes as well as other starchy foods, such as cassava. However, the latter is rarely consumed in Germany and was not assessed in the DEGS1 FFQ. Since EAT-HRD has separate recommendations for fruits, nuts, vegetables and legumes each, we accordingly created four different groups. EAT-HRD gives a joint recommendation for milk and dairy products including cheese expressed as whole milk derivative equivalents. Therefore, we translated intakes of dairy products to their original amount of milk, using conversions factors. These factors were based on the different protein contents of each product as obtained from the German Nutrient Database [32]. As a result, we multiplied food intake of cream cheese with 3, cheese with 7 and cottage cheese/yoghurt/sour milk with 2. Furthermore, meat is separated in red meat and poultry. Since EAT-HDR refers only to added sugars, this group is slightly different as for HEI-MON where free sugars are considered. Free sugars include added sugars and sugars from fruit and vegetable juices [33].

For this index scoring rules also vary if the preferred amount of food intake is plenty, optimal or sparingly (Table 1). For added sugars and red meat, maximum points were awarded for zero intake and points were proportionally subtracted up to the upper recommended threshold. When the upper threshold was exceeded, zero points were given. The EAT-HRD recommends an intake of 0–60% of energy from whole grains and 0–100 g from potatoes. Thus, both recommendations start with zero grams per day. These food groups are major elements of the German diet [34] and are important sources of minerals and dietary fibre, while negative effects on the environment are comparably low [35]. Furthermore, intake of whole grain products is associated with a reduced risk of coronary heart disease, cardiovascular disease, total cancer and mortality from all causes [36]. Therefore, in our index for whole grains, an intake of 15% to 60% of total energy intake received 100 points, for lower and higher intakes points were proportionally reduced until zero points. Since total energy intake could not be properly derived from the FFQ, it was estimated from requirements using standard sex and age specific values for a physical activity level (PAL) of 1, 6. The consumed amount of the specific whole grain foods was converted into calories based on the German Nutrient Database. For potatoes, intakes between 50 and 100 g per day received 100 points, for lower and higher intakes points were proportionally reduced until zero points. In similar indices from other authors, small amounts consumed in these two food groups were also rated with points deducted [18, 19]. According to the EAT-HRD, poultry is interchangeable with eggs, fish, and vegetable protein sources [2]. Therefore, if consumption of fish or eggs exceeded the recommended amount, these excess amounts were counted as part of the consumption of poultry, as long as this consumption was below the recommended amount. For vegetable protein sources, there is no upper consumption limit. Therefore, these consumption levels were not compensated.

### Calculation of summary scores

For both indices, the points of each food group score were summarized and divided by the number of food groups (10 groups for HEI-MON, 12 groups for PHEI-MON). Therefore, both indices range between zero and 100 points. A higher value corresponds to a better dietary/sustainable quality.

Indices were calculated for all persons with a complete set of valid component scores. The HEI-MON was determined for 6758 participants (96.4%) and the PHEI-MON for 6781 (96.7%) participants.

### Statistical analysis

For each index, descriptive statistics were calculated including histograms. Furthermore, mean index values with 95% confidence intervals (CI) were calculated, stratified by sex, age group (18–29, 30–44, 45–64, 65–79 years) and education level (low, medium, high) according to the International Standard Classification of Education (ISCED) [37]. Linear regression models were used to examine the associations between index values and sex, age group and education level with and without simultaneous adjustment.

For every food group, mean intake and mean component score with 95% confidence intervals were calculated. Component scores of 30 and below were rated as low, above 30 and below 60 as medium and of 60 and above were rated as high. The adherence to specific food intake recommendations was evaluated by the proportion of the population that achieved 100 points for those food groups. In addition, the proportion of the population that exceeds the recommended maximum intake of the EAT-HRD was determined as this means transcending the planetary boundaries.

To compare agreement between the two index scores, both were categorized into quartiles. Mean PHEI-MON values for quartiles of HEI-MON and vice versa where calculated. Furthermore, for persons assigned to the respective quartiles of the HEI-MON, the proportional distribution of quartiles of the PHEI-MON were calculated. For both indices, each person was assigned to groups with an index value in one of the low quartiles (one or two) or one of the high quartiles (three or four). This results in an allocation to one of four groups for each person:

- assigned to a high quartile in both indices (group A).
- assigned to a low quartile in both indices (group B),
- assigned to a high quartile in one index and to a low quartile in the other (groups C and D).

For each of these four groups, the mean intake of the food groups was determined. This intake was compared to the mean intake of the total sample. Positive percentage values stand for a higher mean intake in the specific group compared to the total sample, negative percentage values accordingly for a lower intake. Furthermore, the correlation between both index values was calculated using Pearson’s correlation coefficients.

Analyses were conducted with a weighting factor which accounts for the sampling design and corrects sample deviations from the population structure as of 31 December 2010 with regard to age group, sex, region, nationality, community type and education [38]. To consider the weighting as well as the correlation of the participants within a community, the survey procedures for complex samples of SAS 9.4 were used. Level of significance was set at 0.05.

## Results

**Fig. 1:**
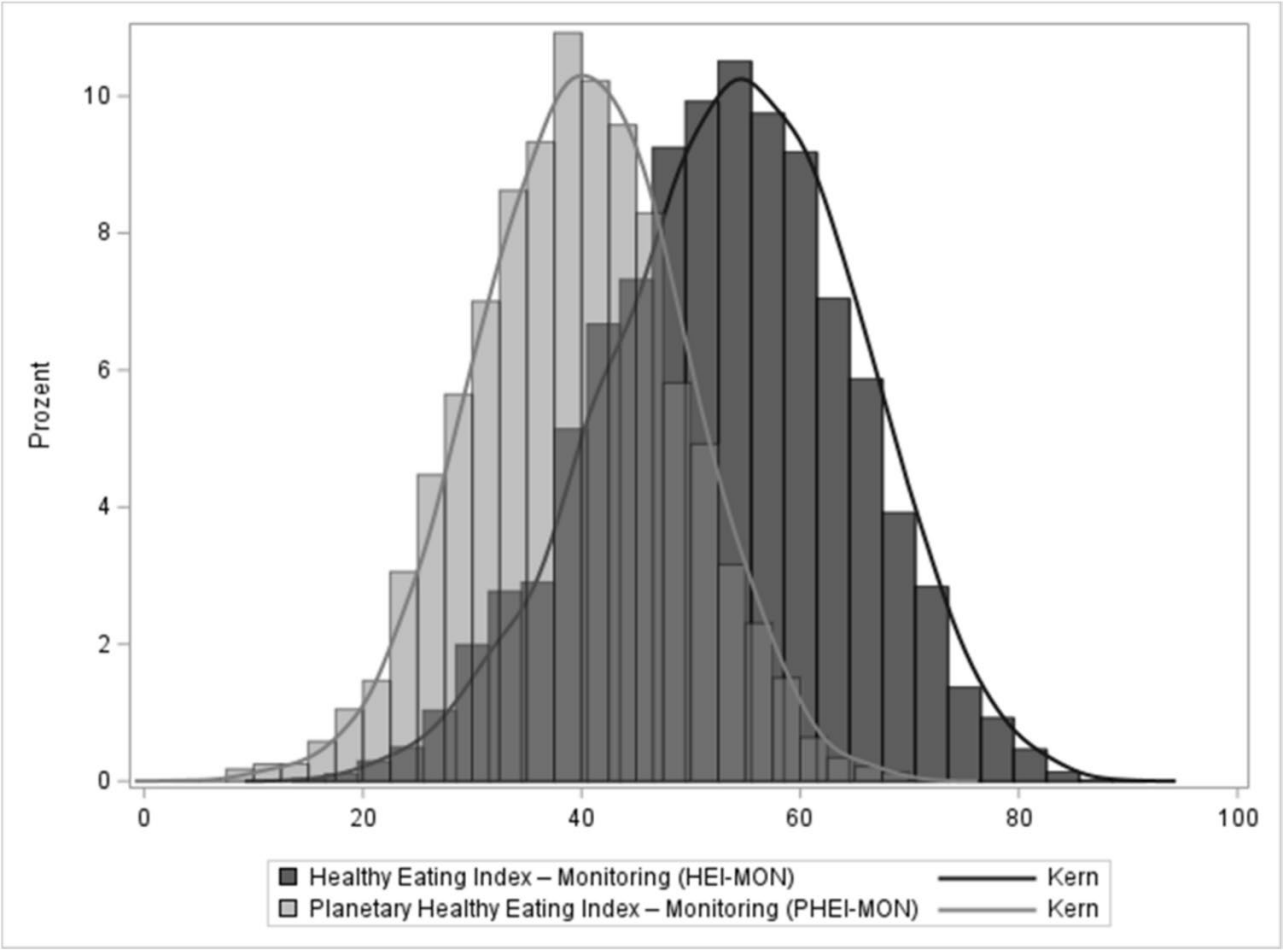
Histogram of the healthy eating index—for monitoring (HEI-MON) and planetary healthy eating index—for monitoring (PHEI-MON), HEI-MON N = 6758, PHEI-MON N = 6781

The mean HEI-MON score was 53 (95% CI 52–53) and the mean PHEI-MON score was 39 (95% CI 39–40), with zero points indicating a poor and 100 points indicating a high correspondence with the respective guideline. Both indices show a fairly normal distribution (Fig. 1).

Women had a higher mean score for both indices than men, e.g. HEI-MON is 54 among women and 51 among men (Table 2). Both indices showed higher mean scores for women and men with higher education and with higher age groups. In linear regression analyses, sex, age group and education level showed independent significant associations with both indices (SI 2).

**Table 2:** Index value of healthy eating index—for monitoring (HEI-MON) and planetary healthy eating index—for monitoring (PHEI-MON) in population groups

			Healthy eating index—for monitoring (HEI-MON)						Planetary healthy eating index—for monitoring (PHEI-MON)						
			N	%^a^	Mean	95% CI		p for trend^b^	N		%^a^	Mean	95% CI		p for trend^b^
Gender	Women^c^		3521	50.4	**54**	54	55	< .0001	3533		50.3	**41**	41	42	< .0001
	Men		3237	49.6	**51**	51	52		3248		49.7	**37**	37	38	
Age in years	Women	18–29	524	18.6	**50**	49	51	< .0001	527	18.6		**38**	38	39	< .0001
		30–44	727	24.5	**52**	51	53		728	24.5		**40**	39	41	
		45–64	1419	36.7	**56**	55	57		1424	36.7		**43**	42	43	
		65-79^c^	851	20.2	**58**	57	59		854	20.2		**43**	43	44	
	Men	18–29	504	19.6	**46**	45	47	< .0001	505	19.6		**34**	33	35	< .0001
		30–44	646	25.7	**49**	47	50		650	25.8		**35**	35	36	
		45–64	1233	37.4	**53**	52	54		1237	37.4		**38**	38	39	
		65-79^c^	854	17.3	**57**	56	58		856	17.3		**40**	39	41	
Education level	Women	High	928	19.8	**58**	56	59	< .0001	930	19.8		**43**	42	44	< .0001
		Medium	2005	55.9	**54**	54	55		2012	55.9		**41**	41	42	
		Low^c^	568	24.3	**52**	51	53		571	24.3		**40**	39	41	
	Men	High	1272	28.6	**54**	53	55	< .0001	1273	28.5		**40**	39	40	< .0001
		Medium	1607	55.4	**51**	50	52		1614	55.5		**37**	36	37	
		Low^c^	342	16.0	**48**	46	49		343	16.1		**34**	33	36	

^a^ Weighted percentage

^b^ Result of linear regression analysis. A p-value < 0.05 was considered as statistically significant

^c^ Reference category

*95% CI* 95% confidence interval

**Fig. 2:**
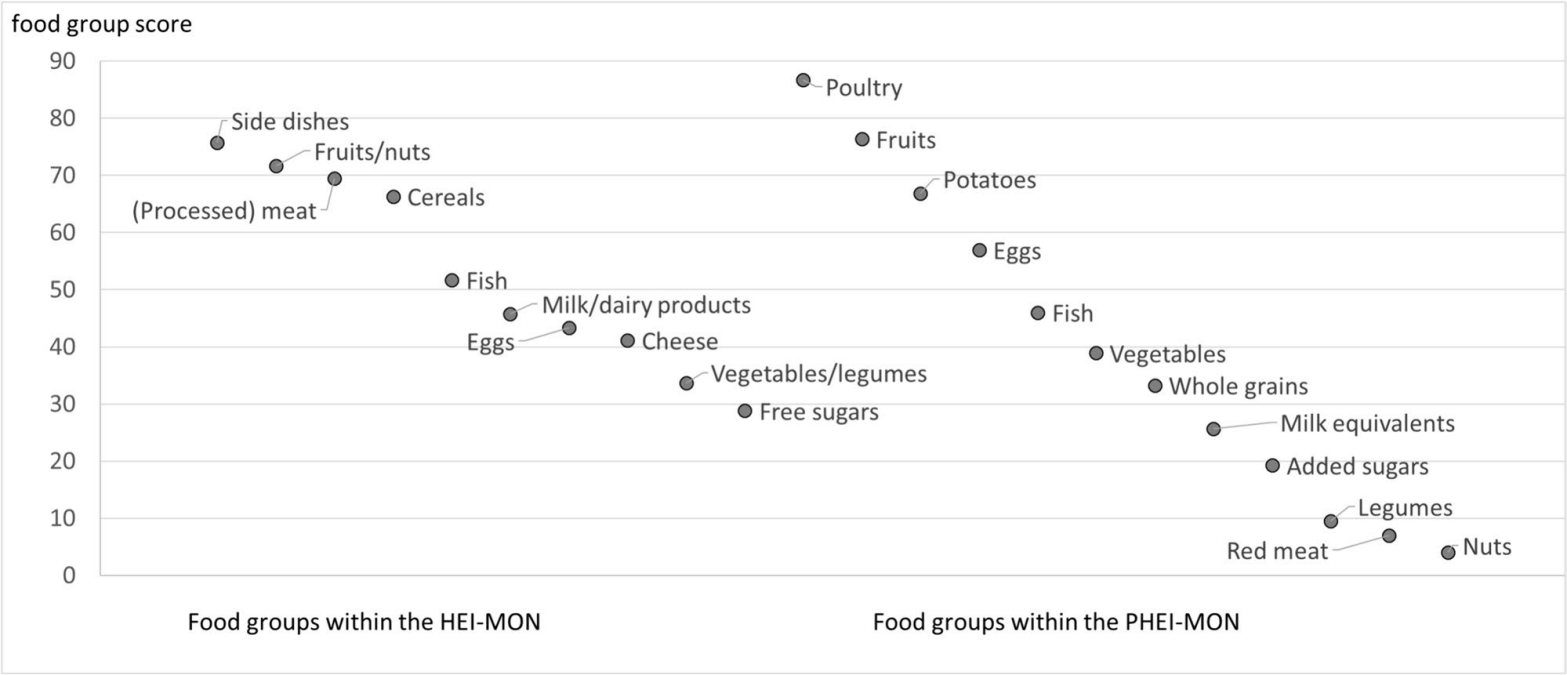
Mean food group scores within HEI-Mon and PHEI-Mon in the German population, HEI-MON N = 6758, PHEI-MON N = 6781

For the food groups, the population’s adherence to the specific guidelines was considerably different. In the HEI-MON, participants achieved highest mean scores for side dishes (76 points), fruits/nuts (72 points), meat/processed meat (70 points) and cereals (66 points) (Table 3, Fig. 2), but scored low for free sugars (29 points). For meat/processed meat intake, 46% of the population achieved the full 100 points which means in this case that they consume less than the upper threshold and the other 54% exceeded the recommended intake. The full score was achieved by 43% for fruits/nuts but only by 4% for vegetables/legumes and by almost nobody (0.1%) for free sugars (Table 3).

**Table 3:** Intake and component score per food group for both indexes (mean with a 95% confidence interval), percentage of the population that completely implements the recommendation (100 points), HEI-MON N = 6758, PHEI-MON N = 6781

	Food group	Intake			Score value			Percentage that achieves 100 points
		Mean	95% confidence interval		Mean	95% confidence interval		
Healthy eating index—for monitoring (HEI-MON)	Cereals (g/day)	**165.9**	161.3	170.5	**66.4**	65.3	67.4	31.4
	Side dishes (potatoes, pasta, rice) (g/day)	**145.9**	142.3	149.6	**75.7**	74.7	76.7	38.7
	Vegetables/legumes (portions/day)	**1.1**	1.0	1.1	**33.7**	32.9	34.5	3.6
	Fruits/nuts (portions/day)	**2.3**	2.2	2.4	**71.7**	70.6	72.7	42.6
	Milk/dairy products (g/day)	**358.8**	342.1	375.4	**45.8**	44.6	46.9	16.1
	Cheese (g/day)	**29.1**	28.0	30.3	**41.2**	40.1	42.2	10.8
	Meat/processed meat (g/day)	**110.4**	107.0	113.9	**69.5**	68.2	70.8	46.1
	Eggs (g/day)	**16.9**	16.1	17.7	**43.3**	42.2	44.4	13.4
	Fish (g/day)	**18.0**	16.8	19.1	**51.7**	50.7	52.8	16.4
	Free sugars (g/day)	**85.7**	81.9	89.5	**28.8**	27.8	29.9	0.1
Planetary healthy eating index—for monitoring (PHEI-MON)	Whole grains (percent of energy intake)	**5.8**^a^	5.5	6.0	**33.3**	32.1	34.5	11.1
	Potatoes (g/day)	**99.4**	95.3	103.5	**66.9**	65.6	68.2	34.3
	Vegetables (g/day)	**125.6**	121.9	129.2	**38.9**	38.0	39.8	8.7
	Fruits (g/day)	**276.4**	267.0	285.8	**76.4**	75.4	77.4	49.3
	Nuts (g/day)	**2.1**	1.9	2.2	**4.1**	3.8	4.4	0.2
	Legumes (g/day)	**13.9**	13.2	14.5	**9.5**	9.1	10.0	0.7
	Milk equivalents (g/day)	**659.5**	637.5	681.6	**25.7**	19.8	21.8	0.1
	Red meat (g/day)	**85.6**	82.4	88.9	**7.0**	6.3	7.7	1.6
	Poultry (g/day)	**24.8**	23.2	26.3	**86.7**	63.8	66.1	66.0
	Eggs (g/day)	**16.9**	16.1	17.7	**56.9**	46.7	49.2	9.1
	Fish (g/day)	**18.0**	16.8	19.1	**46.0**	82.2	83.4	9.8
	Added sugars (g/day)	**66.9**	63.4	70.3	**19.3**	18.3	20.3	0.1

^a^ This corresponds to 130.8 g/day of whole grains (95% CI 125.4–136.3)

**Fig. 3:**
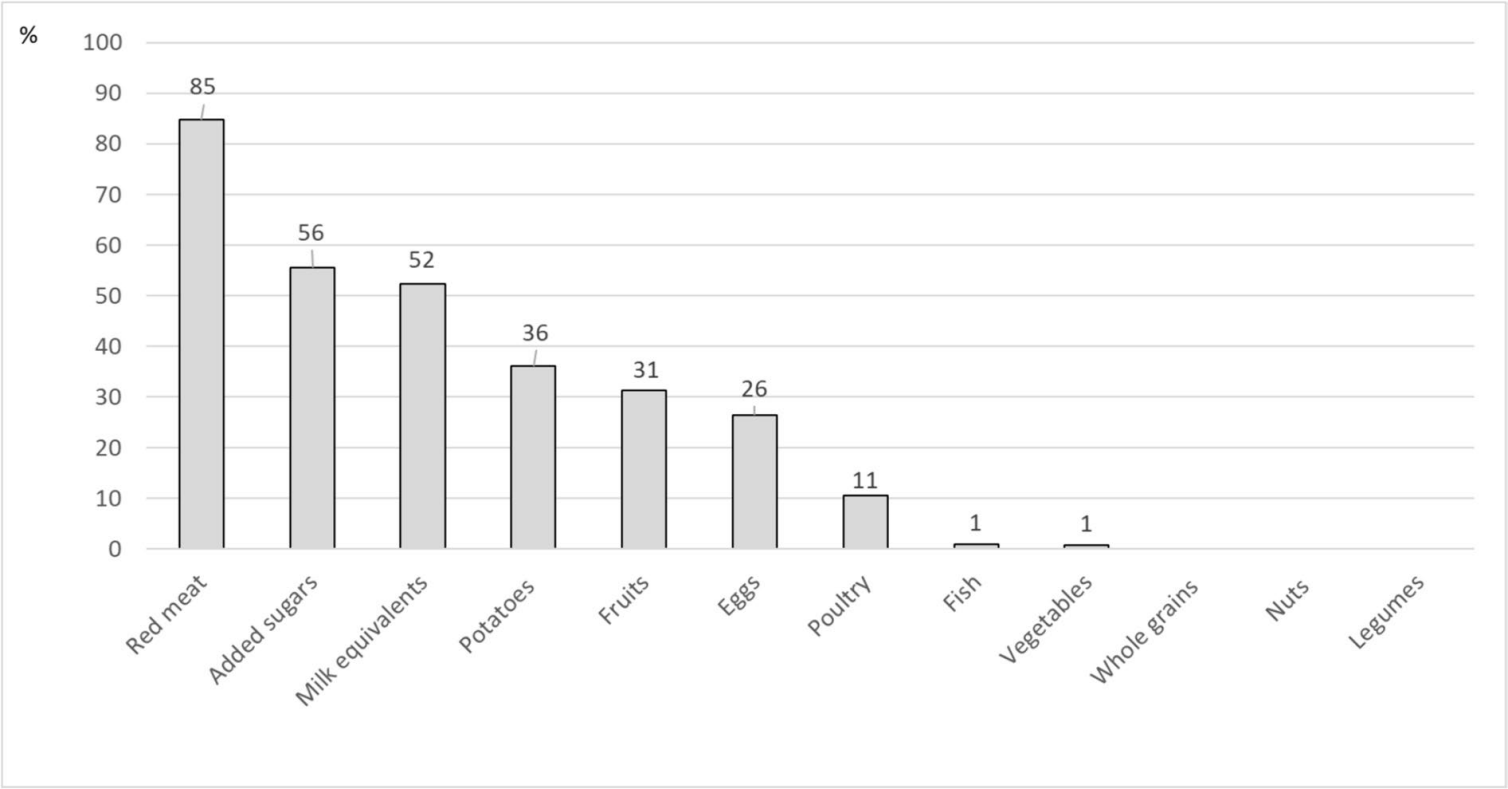
Proportion of the population whose consumption exceeds the maximum recommended amounts within EAT-HRD in percent, N = 6781

For the PHEI-MON, mean component scores were highest for poultry (87 points), fruits (76 points) and potatoes (67 points) (Table 3, Fig. 2). Low mean scores were observed for milk equivalents (26 points) and added sugars (19 points) and very low scores for legumes (10 points), red meat (7 points) and nuts (4 points). The proportion of the population that achieves 100 points is particularly low for nuts, milk equivalents, added sugars, legumes, red meat and eggs (Table 3). The maximum recommended intake is exceeded by many persons for red meat (85% of the population), added sugars (56%) and dairy products (52%) (Fig. 3).

### Comparison of HEI-MON and PHEI-MON

To compare both indices, we grouped persons into quartiles according to both scores. With increasing quartiles of PHEI-MON the mean score of HEI-MON increases (44 (95% CI 43–45), 51 (95% CI 51–52), 56 (95% CI 55–57), 62 (95% CI 61–62)). With increasing quartiles of HEI-MON, the mean score of PHEI-MON increases also (32 (95% CI 31–32), 39 (95% CI 38–39), 42 (95% CI 41–43), 46 (95% CI 46–47)).

**Fig. 4:**
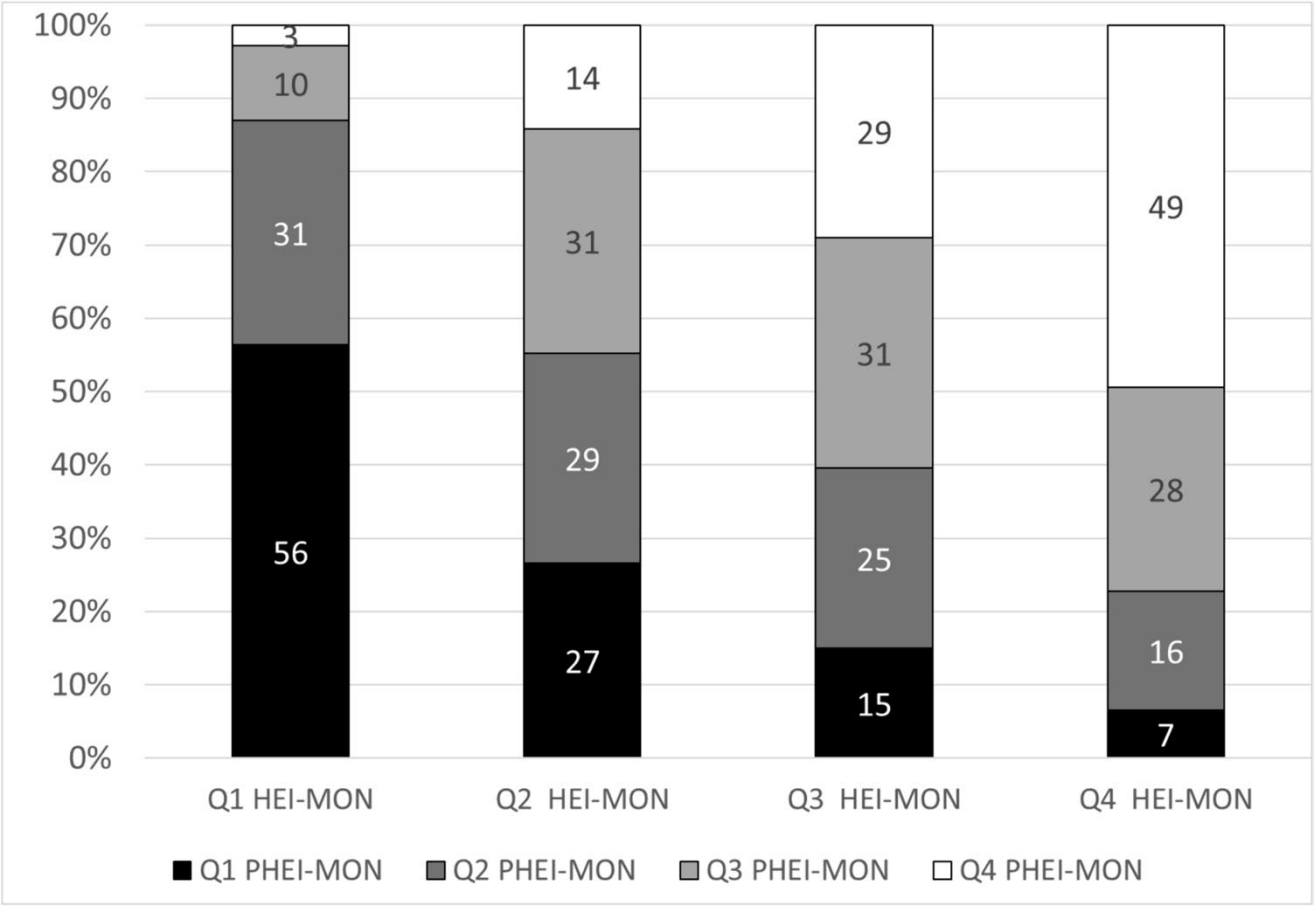
Allocation of study participants to the quartiles of the PHEI-MON in percent, depending on their allocation to the quartiles of the HEI-MON and vice versa, N = 6756. *Q* Quartile, Q1 for the lowest to Q4 for the highest index values

49% of those who eat most in accordance with the German FBDG also agree very strongly with the EAT-HRD (both times in the 4^th^ quartile of the index). Despite very high compliance with the German FBDG (4^th^ quartile in HEI-MON), 7% are assigned to the group with the lowest compliance with the EAT-HRD (1^st^ quartile in PHEI-MON) (Fig. 4).

The individual values of HEI-MON and PHEI-MON are correlated with each other (Pearson’s correlation coefficient was 0.6).

**Fig. 5:**
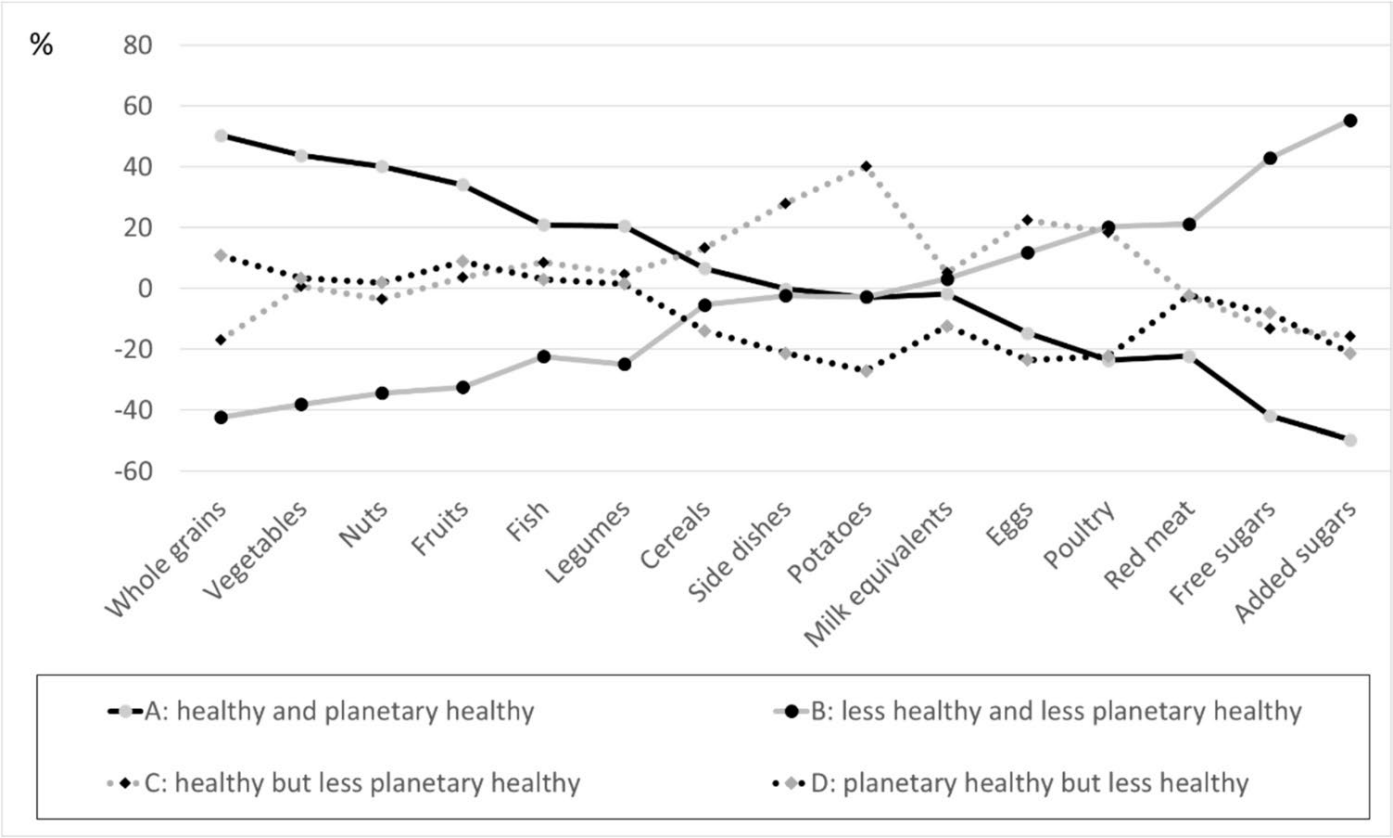
Deviation of the mean consumption in group A–D from the mean consumption of the total population in percent, selected food groups. Persons with the following index scores according to both indexes: Group A: quartile 3 and 4 within both indexes, N = 1.979. Group B: quartile 1 and 2 within both indexes, N = 1.980. Group C: quartile 1 and 2 within PHEI-MON and quartile 3 and 4 within HEI-MON, N = 1.340. Group D: quartile 1 and 2 within HEI-MON and quartile 3 and 4 within PHEI-MON, N = 1.397

High index scores for both indices are achieved by those who consume particularly high amounts of plant foods (whole grains, vegetables, nuts, fruits, legumes) and fish and particularly low amounts of sugar and meat compared to the mean intakes of the total population (Fig. 5, group A). In contrast, people with comparably low intakes of plant foods and fish as well as high intakes of sugar and meat have low index scores for both indices (Fig. 5, group B). High HEI-MON and low PHEI-MON index scores are observed among persons who eat more potatoes/side dishes, eggs and poultry but less whole grains and sugar compared to the total population (Fig. 5, group C). Conversely, people who achieve a high PHEI-MON score but a low HEI-MON score have a relatively low intake of eggs and poultry, potatoes/side dishes and sugar (Fig. 5, group D).

## Discussion

### Summary of main findings

We developed two indices as indicators for adherence to the recommendations of the German FBDG and the healthy and sustainable diet proposed by the EAT-Lancet Commission (EAT-HRD). Applied to nationwide representative food intake data from Germany (2008–2011), the indices showed that a better adherence to either guideline could be found among women, persons of older age as well as persons with higher education level. For both indices, German adults scored particularly high for intake of fruits and potatoes/side dishes. Medium scores were observed for eggs, fish and vegetables, whereas sugar intake often exceeds the amounts recommended by the guidelines, resulting in low scores. There is a linear relationship between both index scores. Nevertheless, at an individual level, the evaluation of the diet may differ substantially depending on the index. Only half of the persons with high scores for the HEI-MON also scored high for the EAT-HRD index. Discrepancies in scoring partly depend on the different rating of some food groups. For instance, different amounts are recommended for dairy products. Furthermore, some food groups are considered separately in PHEI-MON, e.g. fruit and nuts, red meat and poultry. In HEI-MON nuts and fruits are within one group and therefore exchangeable. Overall, people with high scores on both indices are characterized by a relatively high intake of plant foods, such as whole grains, nuts, fruits and vegetables, as well as fish and a particularly low intake of sugar and meat.

### Comparison with other studies

The EAT-HRD uses ranges of recommended intakes, offers some options to exchange certain foods and suggests adaptations to country specific conditions. This makes the scoring criteria and judgement more complex and may lead to different criteria for particular foods in different studies. The scoring system used for the PHEI-MON is similar to the mentioned PHDI [19] in many aspects. However, not all nutritional aspects included in the PHDI could be assessed with the available data for the PHEI-MON. In addition, the PHEI-MON uses slightly different criteria for awarding points, especially for poultry, potatoes and whole grains. Thus, in the PHEI-MON, low consumption quantities of up to 29 g/day for poultry and higher consumption quantities of up to 100 g/day for potatoes do not result in point deductions like in the PHDI. In the case of whole grain, on the other hand, a high proportion of energy from whole grain leads to a deduction of points in the PHEI-MON but not in the PHDI. Therefore, there is no general direction in terms of a stricter or less strict judgement in one of the indices.

Other researchers also constructed indices to quantify the agreement of dietary behaviour with the EAT-HRD for populations in several countries. Although these indices and the assessment methods differ from ours in certain aspects (e.g. type of study, method of food intake assessment, dichotomous or gradual scoring to rate food intake), many studies also described a higher EAT index score for women [14, 19, 22, 24, 25], high education groups [14, 22, 24, 25] and older people [19, 24, 25]. This confirms that measures for dietary improvements should be more appealing to men, low educated and younger people.

A simultaneous analysis of an index based on the EAT-HRD and other traditional indices assessing healthy eating/high diet quality has been performed in cohort studies in Denmark, Brazil and Australia [14, 19, 22]. As they evaluated different indices with different measures of agreement, a direct comparison of the results is difficult. However, similar to our findings, a better score in the EAT-HRD index tended to be associated with a better score concerning healthy eating or a higher diet quality [14, 19], even if the individual correlation measures were sometimes very low [22].

### Strengths and limitations

We introduced two indices based on a comprehensive, detailed scoring system which reflects the consumed amounts of various food items in relation to different dietary recommendations. We assessed the adherence to the diet for both indices, by assigning between 0 and 100 points for intake of different food groups. The indices allow a graduated rating of complex dietary behaviour, enabling us to evaluate the range of adherence in a more nuanced way than dichotomous scores (adherence vs. no adherence). Applied in population-based studies, these indices provide a tangible instrument for evaluating and comparing the degree of compliance with the different dietary guidelines under investigation, also allowing to track changes in eating behaviour over time in an easily understandable manner.

A strength is the data base for the analyses on dietary behaviour. The DEGS study allows for representative statements about the German resident population aged between 18 and 79 years. The food intake data are available for a large study population and were collected with a validated instrument [31]. Even though the survey was conducted some time ago, it provides currently the most recent nationwide data.

When constructing these indices, we had to make a number of decisions, e.g. considering the rating scheme and cut-off points [39, 40]. Some of these decisions were rather subjective. To ensure the transparency of the index construction, the respective decisions were documented. For instance, nuts are rated slightly differently by other authors. This is mainly because of the high-water requirements of certain types of nuts [35], yet their ecological footprint is low compared to several other foods. The partly negative impacts of nuts on the environment may be considered by subtraction of scoring points above a certain consumption level as done previously [13, 23], but not always [19, 25]. However, since nut consumption in Germany is very low, this would have no effect on the PHEI-MON values. The EAT-HRD includes the option that certain foods can be substituted, e.g. poultry with eggs, fish or plant-based protein sources. This may help to ensure that sufficient protein sources are consumed. At present, the protein supply in Germany is widely sufficient [41]. If less animal protein sources are eaten in the future, care must be taken to replace them with plant protein sources.

The assessment of usual consumption frequencies and portion sizes with a self-administered FFQ will results in rough estimates of food intake amounts. Nevertheless, FFQs provide an insight into the usual diet, which is particularly relevant with regard to effects on health. The concept of usual diet fits well to intake recommendations referring to averages over time, which do not have to be reached every single day. However, the quantitative results should be interpreted with caution.

A general limitation of dietary assessment instruments is that they are susceptible to measurement error due to misreporting. The FFQ assesses diet retrospectively, which can lead to recall bias. Social desirability may also influence response behaviour [42, 43]. Compared to other approaches that are used to measure eating behaviour, the FFQ used in our study is relatively short. However, with only very few exceptions, the food groups listed in the recommendations are included. Our FFQ does not include whole grain variants of pasta and rice as well as soy products, which is a drawback given that they are explicitly mentioned in the EAT-HRD. Therefore, we could not include soy products in the PHE-MON index, and the scoring for whole grain products is restricted to bread and cereals, so the complete intake of whole grain products may be underestimated. Furthermore, even though both guidelines give recommendations on fat intake, this was not included, as the FFQ does not provide quantitative information on fat intake. Because of the short FFQ, we neither determined nor adjusted for energy intake, like others have done [18, 19].

The results are based on a survey from 2008–2011. The dietary patterns in Germany may have changed since then, not only because of campaigns for healthy eating, but also because of media coverage and public debate on the climate crisis and sources of greenhouse gases. Climate change is nowadays one of the topics covered most frequently in publications globally [44]. It is conceivable that people have changed their diet over the last years, also to mitigate climate change [45].

### Implications for policy and practice

While the indices are an additional tool for analysing FFQ data and provide a general overview of adherence to guidelines, it is still necessary to look at intake levels of particular foods for targeted policies and interventions. For appropriate policies a regular nutrition monitoring is required and is currently implemented in Germany. Nevertheless, the results of both indices indicate that the average intake of vegetables in Germany does not reach the recommendations. Vegetable consumption would have to be increased about three times in order to meet the recommended amount. It is relevant both for health and the protection of the climate to further promote a higher intake of vegetables. Nuts and legumes are separately mentioned as sources for protein in the EAT-HRD, but are summarized with fruits and with vegetables, respectively, in the German FBDG. They are consumed in small quantities and by only few people in Germany. Nuts and legumes have the potential to reduce cardiovascular and metabolic risks [2]. Interventions that enable individuals to include nuts and legumes as a regular component into their diets, e.g. by adapting menus in (school) canteens, will be relevant for public health.

The German Nutrition Society assumes a higher calcium requirement and recommends a higher intake of milk and dairy products than the EAT-HRD [9]. Still, the intake of milk and dairy products, as measured in our study, is too high even according to the German FBDG. Although milk production in Germany is in global comparison rather efficient due to high milk yields per cow, it still contributes considerably more to greenhouse gas emissions than the production of plant-based foods [46]. Other plant-based sources of calcium could be propagated as an alternative to dairy products, e.g. seeds (poppy, sesame, linseed), amaranth, nuts (almonds, hazelnuts), legumes (chickpeas, tofu), vegetables (kale, broccoli) and algae [32]. However, considering health, it must be noted that milk and dairy products are also important sources for iodine and selenium in addition to calcium. Additional plant-based nutrient sources are needed here, possibly also through fortification with micronutrients, e.g. of plant-based drinks. Otherwise, lower milk intake in the future may result in inadequate nutrient supply, which needs to be monitored.

Both indices also identify dietary components whose intake should be reduced substantially. For sugars, primarily because of its negative impact on health [35], intake should be reduced considerably. On average, meat is consumed more than recommended in the German FBDG, and the differentiated consideration of red meat and poultry according to the EAT-HRD highlights that red meat in particular should be reduced. The average intake of red meat exceeds the maximum recommended amount in the EAT-HRD by factor three. Poultry, on the other hand, is within the range of recommended amount of the EAT-HRD. In recent years, agricultural statistics indicate a decline in meat consumption. This can be observed particularly in the case of pork, where consumption per capita has fallen by an average of 15% since 2012 [47]. But even taking this reduction into account, average red meat consumption would still be about 2.5 times the maximum recommended amount in the EAT-HRD.

Overall, these results underline the need and opportunity to improve the sustainability and healthiness of the German diet. This can make a significant contribution to climate protection through lower greenhouse gas emissions and reduced demand for land and water [2, 19, 48, 49] and also on individual health [2]. To improve communication to the general public, dietary recommendations based on the EAT-HRD should be adapted to local circumstances and may be translated into common units of intake (e.g. portion sizes, usual serving units) and milk equivalents transferred to practical examples of quantities.

Changes in food intake could be achieved, for example, in canteens by changing menus, recipes, and the range of food offered [50, 51] by implementing the DGE quality standards in communal catering, which also include vegetarian and vegan menu lines and are certified sustainable. This could also lead to an increased awareness of the variety of vegetarian or plant-based dishes [52] and could lead to lower production costs in addition to the positive environmental effects [50, 51]. At individual level, information is necessary to counteract concerns regarding meat reduction. In addition, opportunities should be used to improve knowledge about the preparation of plant-based foods [52]. For this purpose, it would be important to explore prevailing barriers and lack of knowledge about a more climate-friendly diet in Germany.

## Conclusions

The analyses show that the health of adults in Germany and the global climate could benefit from a more plant-based diet. This result is similarly evident from the evaluation based on the German Food Based Dietary Guidelines and the Healthy Reference Diet of the EAT-Lancet Commission. The average intake of red meat, milk/dairy products and sugars is too high. At an individual level, the scores for both indices may differ considerably, but overall there is a huge potential in the population to adapt to a diet that is more in line with both guidelines. Recommendations adapted to German eating habits that consider both health and sustainability would therefore be important for the population.

## Supplementary Information

Below is the link to the electronic supplementary material.

Supplementary file1 (DOCX 19 KB)

## Data Availability

The authors confirm that some access restrictions apply to the data underlying the findings. The data set cannot be made publicly available because informed consent from study participants did not cover public deposition of data. However, the data set underlying the findings is archived in the ‘Health Monitoring’ Research Data Centre at the Robert Koch Institute (RKI) and can be accessed by researchers on reasonable request. Requests should be submitted to the ‘Health Monitoring’ Research Data Centre, Robert Koch Institute, Berlin, Germany (e-mail: fdz@rki.de).
