# Supplementary material for: Evaluating the diet in Germany with two indices focusing on healthy eating and planetary healthy eating using nationwide cross-sectional food intake data from DEGS1 (2008–2011)
Source: Eur J Nutr. 2024 Aug 15;63(8):2943–56. doi: 10.1007/s00394-024-03476-x (PMC11519121; doi:10.1007/s00394-024-03476-x)
Supplement: Supplementary file 1 — Supplementary file1 (DOCX 19 KB) [file 394_2024_3476_MOESM1_ESM.docx]

**European Journal of Nutrition**

**Evaluating the diet in Germany with two indices focusing on healthy eating and planetary healthy eating using nationwide cross-sectional food intake data from DEGS1 (2008-2011)**

Almut Richter, Julika Loss, Daria-Alina Kuhn, Ramona Moosburger, Gert B. M. Mensink

Corresponding author: Almut Richter,

Department of Epidemiology and Health Monitoring, Robert Koch Institute, Berlin, Germany

email address: richtera@rki.de

**SI 1** Food groups in the Food Frequency Questionnaire used for the index construction

| **No. Food group** | **No. Food group** |
| --- | --- |
| 1 Milk  2 Sugar sweetened beverages  3 Fruit juice  4 Vegetable juice  5 Fruit, herbal tea (with sub question to sugar content)  6 Black, green tea (with sub question to sugar content)  7 Coffee (with sub question to sugar content)  8 Cornflakes  9 Cereals  10 Wholegrain bread, rolls  11 Brown, rye or mixed bread  12 White bread, rolls  13 Cream cheese  14 Cheese  15 Curd cheese, yoghurt, soured milk  16 Honey, marmalade, jam  17 Nut-nougat cream  18 Eggs  19 Poultry  20 Hamburger, döner kebab  21 Bratwurst, curry sausage  22 Red meat | 23 Sausage  24 Ham  25 Cold fish  26 Fish as a hot meal  27 Fresh fruit  28 Cooked fruit, canned fruit  29 Raw vegetables  30 Legumes  31 Cooked vegetables  32 Pasta  33 Rice  34 Boiled potatoes  35 Fried potatoes  36 French fries  37 Cakes, pies, sweet pastries  38 Biscuits  39 Chocolate, chocolate bars  40 Sweets (such as hard candy, fruit gum, cough sweets, licorice)  41 Ice cream  42 Potato crisps  43 Nuts |

**SI 2** Associations between index values and sex, age group and education level: Results of the linear regression analysis with the index score as dependent variable

a) Healthy Eating Index – for Monitoring (HEI-MON)

|  | | Estimated Regression Coefficients | | | |
| --- | --- | --- | --- | --- | --- |
|  |  | Beta | 95% Confidence Interval | | p-Value |
| sex | male | -3.6 | -4.3 | -2.9 | <.0001 |
|  | female^a^ |  |  |  |  |
| age group | 18-29 years | -9.4 | -10.5 | -8.3 | <.0001 |
|  | 30-44 years | -7.5 | -8.5 | -6.5 | <.0001 |
|  | 45-64 years | -3.6 | -4.4 | -2.8 | <.0001 |
|  | 65-79 years^a^ |  |  |  |  |
| education | high | 5.6 | 4.5 | 6.7 | <.0001 |
|  | middle | 2.7 | 1.8 | 3.7 | <.0001 |
|  | low^a^ |  |  |  |  |

^a^ reference category

b) Planetary Healthy Eating Index – for Monitoring (PHEI-MON)

|  | | Estimated Regression Coefficients | | | |
| --- | --- | --- | --- | --- | --- |
|  |  | Beta | 95% Confidence Interval | | p-Value |
| sex | male | -4.4 | -5.0 | -3.8 | <.0001 |
|  | female^a^ |  |  |  |  |
| age group | 18-29 years | -5.5 | -6.4 | -4.6 | <.0001 |
|  | 30-44 years | -4.2 | -5.1 | -3.3 | <.0001 |
|  | 45-64 years | -1.5 | -2.2 | -0.7 | 0.0003 |
|  | 65-79 years^a^ |  |  |  |  |
| education | high | 3.9 | 3.0 | 4.8 | <.0001 |
|  | middle | 1.6 | 0.8 | 2.4 | 0.0001 |
|  | low^a^ |  |  |  |  |

^a^ reference category
